# Micronutrient deficiencies in heart failure: Mitochondrial dysfunction as a common pathophysiological mechanism?

**DOI:** 10.1111/joim.13456

**Published:** 2022-02-09

**Authors:** Nils Bomer, Mario G. Pavez‐Giani, Niels Grote Beverborg, John G. F. Cleland, Dirk J. van Veldhuisen, Peter van der Meer

**Affiliations:** ^1^ Department of Cardiology University Medical Center Groningen Groningen The Netherlands; ^2^ Robertson Centre for Biostatistics and Clinical Trials University of Glasgow Glasgow UK; ^3^ National Heart & Lung Institute Royal Brompton and Harefield Hospitals Imperial College London UK

**Keywords:** deficiency, heart failure, micronutrients, mitochindrial dysfunction

## Abstract

Heart failure is a devastating clinical syndrome, but current therapies are unable to abolish the disease burden. New strategies to treat or prevent heart failure are urgently needed. Over the past decades, a clear relationship has been established between poor cardiac performance and metabolic perturbations, including deficits in substrate uptake and utilization, reduction in mitochondrial oxidative phosphorylation and excessive reactive oxygen species production. Together, these perturbations result in progressive depletion of cardiac adenosine triphosphate (ATP) and cardiac energy deprivation. Increasing the delivery of energy substrates (e.g., fatty acids, glucose, ketones) to the mitochondria will be worthless if the mitochondria are unable to turn these energy substrates into fuel. Micronutrients (including coenzyme Q10, zinc, copper, selenium and iron) are required to efficiently convert macronutrients to ATP. However, up to 50% of patients with heart failure are deficient in one or more micronutrients in cross‐sectional studies. Micronutrient deficiency has a high impact on mitochondrial energy production and should be considered an additional factor in the heart failure equation, moving our view of the failing myocardium away from an “an engine out of fuel” to “a defective engine on a path to self‐destruction.” This summary of evidence suggests that supplementation with micronutrients—preferably as a package rather than singly—might be a potential therapeutic strategy in the treatment of heart failure patients.

## Graphical abstract

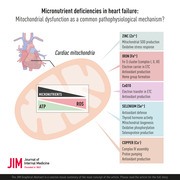

## Introduction

Low plasma concentrations of several micronutrients have been associated with reduced quality of life and adverse outcomes in heart failure (HF) [1, 2, 3, 4]. Most patients with HF consume less than the recommended daily amount of several micronutrients [5, 6], with intake of vitamin D (97% of patients), selenium (95%), zinc (65%) and iron (46%) being most often inadequate [7]. Up to 50% of patients are deficient in one or more micronutrients in cross‐sectional studies [5, 7, 8]. Moreover, patients with HF may have reduced intestinal absorption, increased urinary excretion due to diuretics and defective renal glomerular or tubular function due to oxidative and pro‐inflammatory stress, which exacerbates micronutrient deficiency [5, 9]. Available clinical evidence supports the usefulness of supplementation with some micronutrients to improve HF management in addition to evidence‐based pharmacological therapy [10]. The consensus statement from the Heart Failure Society of America (HFSA) Scientific Statements Committee has already outlined the potential benefit for HF patients when optimizing nutritional status [11]. However, European Society of Cardiology (ESC) HF guidelines do not recommend micronutrient supplementation, other than correcting iron deficiency with intravenous iron [12].

Reduction in bioenergetic capacity plays a major role in the development and worsening of HF [13, 14]. Macronutrients such as fatty acids, lactic acid and carbohydrates are the main energy sources for cardiomyocytes and are consumed in large quantities. Micronutrients—including vitamins, minerals and essential amino acids—are also necessary to convert macronutrients to adenosine triphosphate (ATP), but are required in very small amounts, which a healthy diet normally provides. The mitochondrial electron transport chain (mtETC) requires coenzyme Q10, zinc, copper, selenium and iron for efficient ATP production [15]. Micronutrient deficiency in HF may contribute to defective mitochondrial function and reduced synthetic capacity for ATP.

Despite the identification of a high prevalence of micronutrient deficiencies in HF and their association with an adverse prognosis, with the exception of iron, very few randomized trials of micronutrient interventions have been conducted [16]. Therefore, a little information is provided on micronutrients in HF management guidelines [17, 18]. In this review, we focus on five key micronutrients involved in mitochondrial ATP production (Table 1).

**Table 1:** Five key micronutrients involved in mitochondrial adenosine triphosphate (ATP) production and deficiency in heart failure

Micronutrient	Physiological role	Role in the ETC	Prevalence of deficiency in HF	Associations with deficiency in HF	Clinical response to supplements in HF
Iron	Production and function of hemoglobin and myoglobin and therefore oxygen uptake and transfer DNA replication and repair, lipid metabolism and chromatin modification[19]	Enabling oxidative phosphorylation by ATP synthase within the mitochondria [20]	37%–61% [2, 21, 22, 23]	Worsening symptoms, quality of life, functional status and clinical outcomes, including mortality, irrespective of ejection fraction [21, 22, 24–26]	Substantial trials in patients with HF [27] Alleviates symptoms and improves exercise capacity and quality of life, reduces HF hospitalizations, but uncertain effect on mortality [27, 28, 29, 30, 31] Beneficial effects on peak VO_2_ compared with standard of care treatment [30]
Selenium	Bioactivity of (local) thyroid hormone [32, 33, 34] Promoting anti‐inflammatory cytokine expression and controlling the immune response [39]	Crucial antioxidant (redox) enzymes [35, 36]	20% [37]	Severe selenium deficiency in humans may cause a dilated cardiomyopathy (Keshan disease) [38] Impaired exercise tolerance, reduced quality of life and higher mortality rate [37]	No large RCTs in patients with HF Combination of CoQ10 and Se in older people, many of whom had HF, reduced cardiovascular mortality [40]
Zinc	Growth, reproduction and immune system [41]	Antioxidant defenses by superoxide dismutase (Cu/Zn‐SOD) [42, 43]	66% [3]	Increased risk of cardiovascular and all‐cause mortality [3]	No large RCTs in patients with HF
	Synthesis and degradation of carbohydrates, lipids, proteins and nucleic acids [41]			Increased NYHA functional class, older age and use of ACE inhibitors and angiotensin II receptor blockers [3, 44]	Combination of zinc and Se improved left ventricular ejection fraction [45]
	Catalytic activity of ACE [46]			Increased inflammation and myocardial damage (C‐reactive protein and troponin I), and impaired exercise capacity [3]	Multi‐micronutrient supplements increased LVEF/LVEDV [47, 48]
Copper	Immune function [49]	Mitochondrial electron transport and free radical scavenging by Cu/Zn‐SOD [50, 51, 52]	NR	Problems with connective tissue, muscle weakness and anemia [53] Compromised cardiac mitochondrial respiration and impaired ATP production [55]	Ongoing RCT [54]
CoQ10	Inhibiting the peroxidation of lipids and lipoproteins [56]	Redox reactions within the electron transport chain, regulating ATP production [57] Facilitating electron transfer from complexes I and II to complex III [58, 59]	59% [60]	Increased NYHA functional class, lower LVEF and increased NT‐proBNP levels [61, 62]	Symptom relief, increases exercise‐capacity, duration, peak oxygen consumption and quality of life [6] Improves symptoms, and reduces major adverse cardiovascular events [63]
					Combination of CoQ10 and Se in older people, many of whom had HF, reduced cardiovascular mortality [40]

Abbreviations: ACE, angiotensin‐converting enzyme; ATP, adenosine triphosphate; CV, cardiovascular; ETC, electron transport chain; HF, heart failure; LVEDV, left ventricular end‐diastolic volume; LVEF, left ventricular ejection fraction; NR, not reported; NYHA, New York Heart Association; RCT, randomized controlled trial; SOD, superoxide dismutase.

## Mitochondrial dysfunction as common denominator

**Fig. 1:**
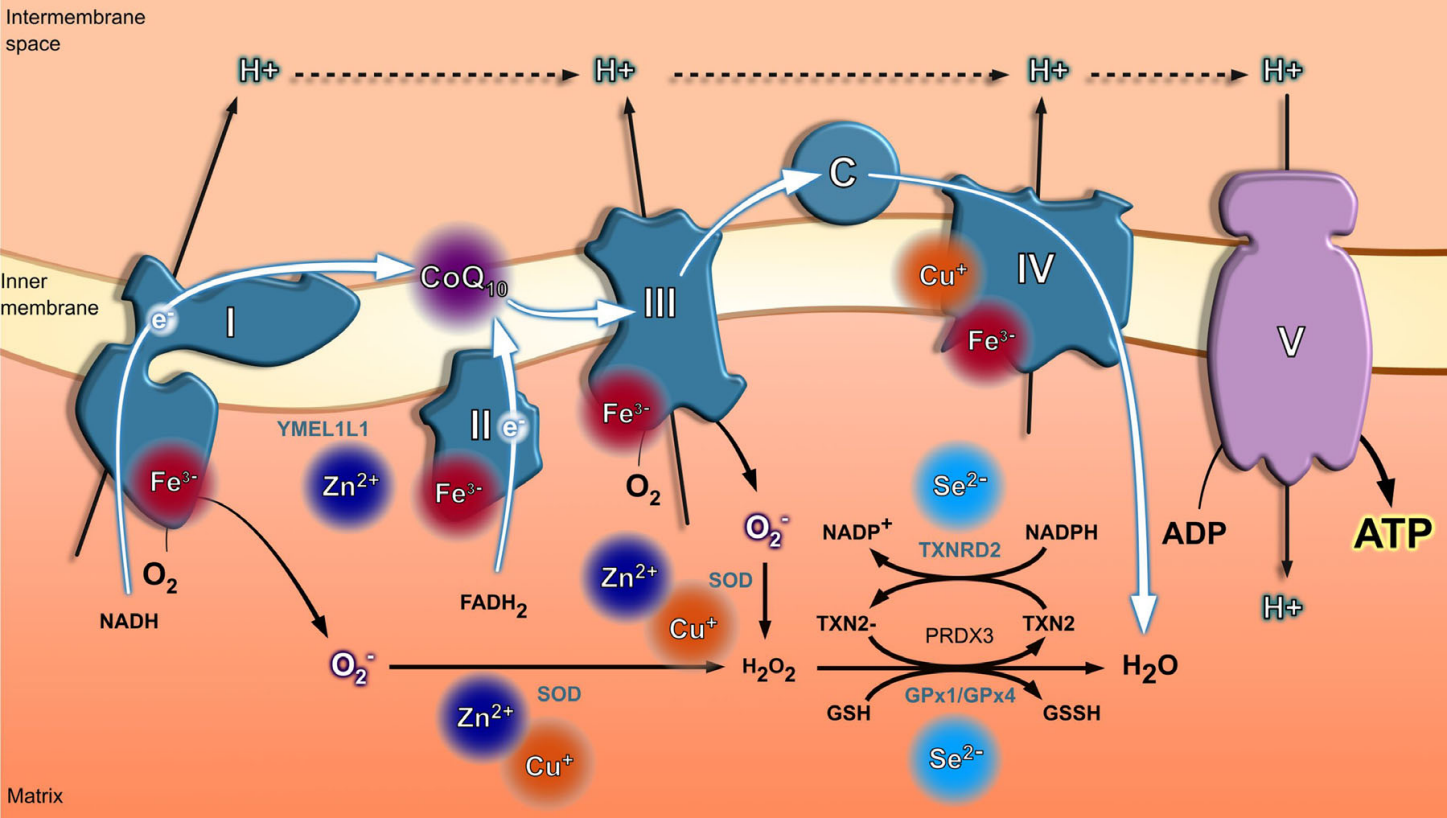
Micronutrients in the mitochondrial electron transport change (mtETC). The electron transport chain (ETC) starts with a proton transfer (H^+^) mediated by complexes I and II, which promotes an electrochemical gradient across the mitochondrial membrane. Complex III (ubiquinol‐cytochrome c oxidoreductase or CIII) forms the central part of the mitochondrial respiratory chain, oxidizing CoQ10 and reducing cytochrome c while pumping protons from the matrix to the intermembrane space through the so‐called Q‐cycle mechanism. Finally, four cytochrome C molecules deliver an electron to complex IV (cytochrome c oxidase or CIV), being carried by the complex and transfer them to one dioxygen molecule, converting the molecular oxygen to two molecules of water. The electrochemical gradient is used by complex V (adenosine triphosphate [ATP] synthesis) to promote the generation of ATP from the available adenosine diphosphate (ADP). Although the ETC is a quite efficient mechanism to promote energy formation, the proton gradient generation results in an elevated reactive oxygen species (ROS) production due the O_2_ oxidation into O_2_^−^ (superoxide anion radical), H_2_O_2_ and OH (hydroxyl radical), which are the toxic products of respiration. Micronutrients present a key role in the proton gradient generation (CoQ10) and electron carrier transfer among the different complexes (Fe^3+^ and Cu^+^). Furthermore, Cu^+^, Zn^2–^ and Se^2−^ participate in the oxidant scavenger system, decreasing toxic mitochondrial ROS. Abbreviations: FADH2, flavin adenine dinucleotide; GPXs, glutathione peroxidases; GSH glutathione; reduced NADH, nicotinamide adenine dinucleotide; PRDX3, peroxiredoxin 3; SOD, superoxide dismutase; TXN2, thioredoxin 2; TXNRDs, thioredoxin reductases.

Micronutrients serve as cofactors or part of essential amino acids in enzymatic reactions because they facilitate the transition between oxidized and reduced states. The systemic and cellular levels of some micronutrients—iron and copper in particular—must be balanced, as deficiency leads to loss of activity of crucial enzymes, but their accumulation may be toxic. Micronutrients are particularly important for normal mitochondrial function, especially in mitochondria‐rich tissues such as the myocardium. Oxidative phosphorylation is the process by which ATP is formed by the transfer of electrons from 1,4‐dihydronicotinamide adenine dinucleotide (NADH) or dihydroflavine‐adenine dinucleotide (FADH_2_) to oxygen (O_2_) by a series of electron carriers [64]. Mitochondrial oxidative phosphorylation is responsible for approximately 90% of the ATP produced in cardiomyocytes. An abundance of iron (complexes I, II, III and IV), selenium (redox and antioxidant), zinc (mitochondrial antioxidant), copper (complex IV) and coenzyme Q10 (CoQ10) (complexes I, II and III) is essential for the normal function of the mtETC (Fig. 1).

## Iron

Iron is essential for oxygen transport and ATP production, but an excess of nonferritin‐bound iron in cells is potentially extremely toxic. Iron accepts and donates electrons, switching between its bivalent (Fe^2+^) and trivalent (Fe^3+^) form [65], leading to formation of reactive oxygen species (ROS). Cells require protection from free iron, provided by ferritin intracellularly and transferrin in the plasma [25, 66]. Most of the body's iron is bound to hemoglobin or myoglobin, or it is stored intracellularly bound to ferritin or—especially in macrophages and the liver—bound to hemosiderin. A much smaller amount of iron is also found bound to carrier proteins such as transferrin or to nonheme enzymes involved in oxidation–reduction reactions and the transfer of electrons (cytochromes and catalase) in mitochondria [65]. The ability of iron to engage in redox reactions makes it a widely utilized cofactor in many key biochemical processes including oxygen delivery and storage, mitochondrial oxidative phosphorylation, DNA replication and repair, lipid metabolism and chromatin modification [19]. Specialized transporters, mitoferrin 1 and 2, move iron from the cytoplasm into the mitochondria [67]. Mitochondria play a key role in iron metabolism in which they synthesize heme, assemble iron–sulfur (Fe/S) proteins and participate in cellular iron regulation [68]. Iron is directly involved in several complexes of the mtETC, containing Fe/S clusters (complexes I, II, III and IV) and heme (complexes II, III and IV), enabling oxidative phosphorylation by ATP synthase within the mitochondria [69], but iron is also a critical component of many other heme‐containing enzymes involved in energy production [70]. Activity of mitochondrial complexes I–III, which predominantly contain Fe/S clusters, was shown to be adversely affected by deferoxamine (DFO) treatment, while the exclusively heme‐based complexes IV and V showed unaltered activity (Hoes et al. [20]). These findings were confirmed by Khechaduri et al. [71], who concluded that despite global mitochondrial dysfunction, heme levels were maintained above baseline in human failing hearts, and Melenovsky et al. [72], who also showed a decreased activity of mitochondrial complexes I and III in HF patients. However, the underlying mechanism regulating heme conservation remains unclear.

Several in vitro studies have shown detrimental effects of iron deficiency on mitochondrial function and morphology in human cardiomyocytes and myoblasts [20, 72, 73]. Iron deficiency provokes a hypoxic response, resulting in mitochondrial dysfunction, reduced ATP production and impaired contractility and relaxation of cardiac myocytes [20]. Iron‐deficient mitochondria can also lead to the production of oxygen radicals (O_2_^−^) by complexes I and III [74], causing injury to mitochondria and other organelles. Adding transferrin‐bound iron to these cells can correct these abnormalities [20]. It needs to be recognized that the circulatory, cellular and organellar systems covering iron deficiency might be subjected to separate and different regulatory mechanisms. For example, differentiating between cytosolic and mitochondrial iron content showed that mitochondrial iron was actually increased, while cytosolic iron was reduced [71].

Human mitochondrial function in patients with HF and iron deficiency has been assessed in skeletal muscle using phosphorus‐31 magnetic resonance spectroscopy (^31^P MRS). This technique allows for noninvasive, real‐time detection of phosphocreatine (PCr), inorganic phosphate and ATP. PCr comprises a pool of chemical energy, rapidly available during exercise, which gets replenished during periods of rest. Impaired skeletal muscle energetics—often accompanied by lower pre‐exercise PCr levels, a lower pH and a slower recovery rate of PCr after exercise—are observed both in patients with Heart Failure with reduced Ejection Fraction (HFrEF) and Heart Failure with preserved Ejection Fraction (HFpEF) [75, 76, 77]. Among patients with iron deficiency, these abnormalities are more pronounced [77]. Administration of a single dose of iron isomaltoside in a randomized, double‐blind, placebo‐controlled trial including 40 patients with HFrEF improved PCr recovery within a few weeks [78]. Future studies into organ‐, cell‐ and organelle‐specific iron regulation and pathophysiology will hopefully provide further insights [79].

Iron deficiency is the most common nutritional deficiency, estimated to affect more than 2 billion people worldwide [80]. Gastrointestinal absorption of iron is inefficient: Only 5%–10% of iron is actually taken up, and may be reduced by many factors including diet, inflammation and edema. Most patients with HF have or will develop iron deficiency [2, 21–23]. Patients with HF and iron deficiency have worse symptoms, quality of life, functional status and clinical outcomes—including mortality—irrespective of left ventricular ejection fraction (LVEF) [21, 22, 23, 24, 25, 26].

Several substantial, randomized, double‐blind, placebo‐controlled multicenter trials investigating intravenous (IV) iron—mainly with ferric carboxymaltose—in patients with HF and a reduced LVEF (HFrEF) have shown beneficial effects on symptoms, quality of life and exercise capacity [29, 30, 31]. Similar evidence does not exist for oral iron, possibly because of ineffective iron uptake and/or lack of sufficient robust trials [81, 82]. The most recent ESC guidelines for the diagnosis and treatment of acute and chronic HF recommend that all patients with HF should be tested for iron deficiency, and treatment with IV iron should be considered to improve symptoms, exercise capacity and quality of life [83]. The 2017 American Heart Association/American College of Cardiology (AHA/ACC) guidelines made similar recommendations [84]. Recently, results were published from the AFFIRM‐AHF trial (*n* = 1038), which investigated the effects, compared with placebo, of IV ferric carboxymaltose administered prior to discharge to patients who had been admitted with acute HF, an LVEF less than 50% and iron deficiency using the FAIR‐HF trial criteria. Patients assigned to IV iron had fewer rehospitalizations for HF, but no effect on mortality was observed [28]. Iron deficiency is also common in patients with HF and an LVEF greater than 40% (HFpEF), especially for those with more severe diastolic dysfunction, and is associated with poorer exercise capacity and quality of life [85]. An updated meta‐analysis of randomized trials including patients with iron deficiency and HFrEF suggested that IV iron reduced hospitalization for HF by about 30% (odds ratio [OR]: 0.67 [0.54–0.85]; *p* = 0.0007) but could not confirm an effect on cardiovascular mortality (OR: 0.89 [0.66–1.21]; *p* = 0.47) [27]. This meta‐analysis does not exclude the possibility of a substantial reduction in mortality with IV iron, although neither does it rule out a modest increase. Several more studies are to be completed in the next 2 years. Currently, data are lacking on the effects of iron supplementation in patients with HFpEF.

## Selenium

Selenium is a component of selenocysteine [86], an amino acid that is required for the formation of selenoproteins [87, 88], including glutathione peroxidases (GPXs), thioredoxin reductases (TXNRDs) and iodothyronine deiodinases (DIOs). The function of these selenoproteins in the heart is not understood completely [1, 89–91]. Proteins from the GPX and TXNRD families are crucial antioxidant (redox) enzymes involved in preventing the harmful accumulation of intracellular hydrogen peroxide, including in the mitochondria [35, 36]. DIOs regulate the local bioactivity of thyroid hormone, which stimulates mitochondrial biogenesis, increasing myocardial mitochondrial mass, mitochondrial respiration, oxidative phosphorylation, enzyme activities, mitochondrial protein synthesis, cytochrome, phospholipid and mitochondrial DNA (mtDNA) content [32, 92].  The human selenoproteome also includes methionine sulfoxide reductase B1 (MSRB1), which promotes anti‐inflammatory cytokine expression and controls the immune response [39]. Two other selenoproteins are crucially involved in selenoprotein synthesis (selenophosphate synthetase 2 [SEPHS2]) and transport (selenoprotein P [SELENOP]) [34, 93]. SELENOP is of special interest, since it was shown to correlate very well with selenium levels [94]. SELENOP is the main protein responsible for carrying selenium in the circulatory system [91]. In the context of acute HF, it has been reported that patients with low SELENOP levels had greater risk for 30‐day rehospitalization (hazard ratio [HR]: 4.29; 95% confidence interval [CI]: 1.59–11.6), 1‐year mortality (HR: 4.13; 95% confidence interval [CI]: 1.64–10.4) and a composite endpoint of death or rehospitalization within 30 days (HR: 4.80; 95% CI: 1.80–12.8) compared with patients with higher levels [94]. Similarly, in a large Swedish prospective cohort study in the general population, patients with high SELENOP levels have significantly lower risk for all‐cause mortality (0.57, 0.48–0.69), cardiovascular mortality (0.52, 0.37–0.72) and first cardiovascular event (0.56, 0.44–0.71) [93]. The importance of other selenoproteins is uncertain [86]. Selenium deficiency impairs the ability to synthesize selenoproteins, increases oxidative stress, reduces the response to thyroxine and impairs the immune responses [95, 96]. Two well‐described and long‐known hierarchical principles can be observed in selenium availability and usage [97]. Not all tissues and selenoproteins are equally well supplied with the trace element when limited. First, selenium concentrations in the different tissues were found to vary, and selenium preferentially accumulated, and was retained efficiently, in specific organs such as the testes, adrenals and the brain. Second, comparisons of selenoprotein dependence on selenium status showed that specific selenoprotein transcripts compete for the limited amount of selenium and decrease gradually in response to deficiency [97]. Nevertheless, knowledge on the hierarchical position of the heart among other organs, and the cardiac‐specific selenoprotein hierarchy, is limited.

Selenium deprivation in human pluripotent stem cell (PSC)‐derived cardiomyocytes impairs mitochondrial respiration, biogenesis and oxidative stress [37], which may reflect the production of inactive GPX enzymes and subsequent impaired usage of glutathione [98, 99, 100]. ROS are increased in selenium‐depleted cardiomyocytes under normal culture conditions [37], which can be corrected by restoring selenium [37]. Cell apoptosis caused by reperfusion injury, which is mediated by oxidative stress, may also be exacerbated by selenium deficiency [101].

Severe selenium deficiency in humans may be associated with a rare but fatal form of dilated cardiomyopathy (DCM)—Keshan disease—that was reported from a specific geographic region where there was a very low amount of selenium in the soil and therefore in food [38]. Keshan disease is reversible with selenium supplementation [1]. Selenium soil content is very variable. For example, intakes are high in Venezuela, Canada, the United States and Japan (>100 μg/day), and much lower in some parts of Europe (∼40 μg/day) [95]. Observational studies in the general population [95, 102] suggest that selenium deficiency might be common, but data for patients with HF are scarce [47, 103, 104]. Recently, selenium deficiency (serum selenium <70 μg/L) was found to be associated with reduced exercise capacity and a substantially higher mortality in a prospective cohort study [37]. Furthermore, serum selenium concentrations of 70–100 μg/L were associated with similar adverse findings [37], suggesting that values below 100 μg/L might be considered to indicate deficiency [105, 106]. Selenium (<100 μg/L) and iron deficiency may often coincide [107], although, unlike iron, selenium is readily absorbed orally.

No substantial randomized trial of selenium supplements has yet been done in patients with HF. One placebo‐controlled, randomized trial (*n* = 443) investigated the effects on cardiovascular mortality of supplements of selenium together with CoQ10 given for 4 years to older Swedish citizens, some of whom had HF (KiSel‐10 study; *n* = 443; age 70–88 years) [108]. Despite discontinuing supplements after 4 years, cardiovascular mortality was lower at 10 years in those who had been assigned to supplementation (HR: 0.51; 95% CI: 0.36–0.74; *p* = 0.0003). Although most patients had cardiovascular problems, only a minority had HF. Additionally, one very small randomized controlled trial (RCT) has been performed in patients with HF from Iran. Garakyaraghi et al. [109] supplemented 32 patients with congestive heart failure (CHF) with a combination of 90 mg CoQ10 and 200 μg selenium per day for 3 months. This led to favorable effects on New York Heart Association (NYHA) classification, LVEF and myocardial performance index compared to the placebo group [109]. Besides the low number of included subjects, it cannot be concluded whether these effects are a result of selenium supplementation or CoQ10 use as they were given coincidentally, especially as no serum levels of either were measured. A meta‐analysis of nutrients with potential antioxidant properties suggested that only supplements that included selenium reduced cardiovascular disease (CVD) mortality (relative risk [RR]: 0.77; 95% CI: 0.62–0.97; *p* = 0.02) [110], even though the KiSel‐10 trial was not included. Supplements of other antioxidants—including vitamins A, C, and E, β‐carotene and retinol—were not associated with a lower mortality. Nonetheless, there is uncertainty and a paucity of robust data [111, 112, 113]. Only patients with selenium deficiency might benefit from supplements [98, 110]. Molecular evidence suggests that a serum selenium >100 μg/L is required for optimal GPX activity [34, 114–117]. A meta‐analysis that stratified studies into regions with higher (North‐ and South‐America) or lower (Europe and Asia) soil selenium content suggested a greater reduction in mortality with selenium supplements when soil selenium content was low (relative risk [RR]: 0.88; 95% CI: 0.78–0.98; *p* = 0.02), but a possible increase in mortality when selenium soil content was high (RR: 1.06; 95% CI: 1.01–1.12; *p* = 0.03) [118]. The hypothesis that only patients who have evidence of selenium deficiency benefit from supplements is plausible but should be evaluated in a well‐designed clinical trial.

## Zinc

Zinc is vital for many physiological functions, including growth, reproduction, antioxidant defenses and the immune system. It is a critical component of the catalytic site of more than 300 enzymes and required for the synthesis and degradation of carbohydrates, lipids, proteins and nucleic acids [41]. Zinc also influences neuronal function and hormone release [119]. Angiotensin‐converting enzyme (ACE)—a membrane metallopeptidase that requires zinc for its catalytic activity [120]—plays a key role in the production of angiotensin II and degradation of bradykinin and many other vasoactive peptides [46]. Additionally, other Zn‐dependent proteases such as aminopeptidases are also known to limit and modulate angiotensin function, showing the importance of zinc sufficiency in the proper regulation of the ACE‐associated pathways [121]. ACE inhibitors, angiotensin II receptor blockers and thiazide diuretics may all reduce serum zinc concentrations [122].

The World Health Organization (WHO) suggests that zinc deficiency is common in many regions of the world [123]. Zinc deficiency can be caused by a diet high in phytate‐containing whole grains—as phytate is a potent ligand for zinc that prevents absorption [124]—foods grown in zinc‐deficient soil, or processed foods containing little or no zinc [125]. Plasma or serum zinc concentrations may be a poor indicator of moderate, subclinical zinc deficiency [124]. In humans, zinc deficiency usually occurs in the context of deficiencies of other nutrients, such as iron.

Mitochondrial peroxiredoxin and metalloenzymes, such as superoxide dismutase (SOD), rely on zinc for antioxidant reactions [126]. When mitochondrial production of ROS exceeds the rate of restoration of antioxidant defenses by zinc‐dependent superoxide dismutase (Cu/Zn‐SOD), mitochondrial permeability is increased, leading to swelling and degeneration and, ultimately, cell death [42, 43]. Next to the activity of SOD, zinc is also involved in the dynamic control of mitochondrial activity through so‐called mitochondrial proteases. The ATP‐dependent zinc metalloprotease YME1L1 is probably the best‐described mitoprotease and was shown to be involved in mitochondrial protein quality control, mitochondrial biogenesis, mitochondrial stress responses, mitochondrial dynamics, mitophagy and apoptosis [127]. Nevertheless, the direct functional link between this protease and zinc deficiency in the heart is not yet established. When chronic inflammation is present, zinc deficiency may contribute to apoptosis and myocardial necrosis [128]. Exogenous zinc regulates the activities of several key intracellular signaling elements such as mammalian target of rapamycin (mTOR), extracellular signal‐regulated kinase (ERK) and glycogen synthase kinase‐3β (GSK‐3β). Zinc‐induced inactivation of GSK‐3β inhibits mitochondrial permeability transition pore (mPTP) opening, preventing reperfusion injury [129].

Animal studies suggest that zinc supplementation may protect against loss of systolic function and enhance diastolic function during and after ischemia‐reperfusion injury [130]. Zinc might reduce oxidative stress partly by enhancing the activity of the zinc‐binding protein, metallothionein [41, 131], leading to increased zinc uptake and release in cardiac tissue [130, 132, 133]. Furthermore, zinc deficiency might also play a role in the development of a cardiomyopathy that can be reversed by zinc supplements [41, 45, 134, 135].

In HF, zinc deficiency may be due to low dietary intake, reduced gastrointestinal absorption, and/or increased excretion as the result of neurohormonal activation [7, 135–137]. Metabolic stress may increase cellular uptake of zinc to regulate antioxidant defenses. ACE inhibitors and angiotensin II receptor blockers (ARBs) increase urinary and fecal zinc excretion [137]. Although the prevalence of zinc deficiency in patients with HF is uncertain, serum zinc concentrations have been reported to be lower than those reported as normal values for healthy volunteers (75–140 μg/dl) [44, 45, 135, 138]. A prospective observational study of 1079 patients with decompensated HF suggested that serum zinc concentrations were less than 75 μg/dl in 66% of patients and that such values were associated with a higher cardiovascular and all‐cause mortality [3]. Amongst patients with HF, serum zinc concentrations decline with worsening NYHA class, older age, and use of ACE inhibitors and ARBs [3, 44]. Low serum concentrations are also associated with hyponatremia, iron deficiency, increases in C‐reactive protein (CRP) and troponin I—suggesting inflammation and myocardial damage, respectively—and with impaired exercise capacity [3].

There is a paucity of evidence to support zinc supplements. In a small, non‐randomized, prospective study, 10 patients with malabsorption‐associated cardiomyopathy received zinc and selenium supplements (Addamel N, 10 ml given intravenously; corresponding to 300 μg selenium and 13.6 mg zinc every day for 1 week, and subsequently every week for 6 months) [45]. Eight patients with malabsorption‐associated cardiomyopathy served as controls. Monthly injections increased serum and myocardial zinc content and LVEF (from 28% to 42%; *p* < 0.001), which were associated with clinical improvement. In myocardial biopsies, the content of selenium and zinc and GPX activity increased, with striking improvements in cardiomyocyte degenerative changes including a reduction of cell autophagy and myofibrillolysis [45]. A recent observational study evaluated the role for zinc supplements in patients with nonischemic heart disease. Patients (*n* = 25) were given zinc acetate—50 mg three times a day for 10 months—and were compared with 10 healthy subjects on similar treatment. However, the results of these studies have not yet been published [139]. Recently, a case report was presented of a 24‐year‐old female who was diagnosed with anorexia nervosa and new‐onset HF [140]. Supplementing her with oral zinc (220 mg/day), together with guideline‐recommended HF therapy and anorexia‐nervosa management, resulted in improved left ventricular systolic function. This case was pointed out as the first report of low plasma zinc levels as the probable cause of cardiomyopathy, which improved after zinc supplementation. Again, well‐designed clinical trials of sufficient size enrolling patients with HF and zinc deficiency are required to understand the potential therapeutic role of zinc supplements.

## Copper

There are many copper‐dependent proteins (known as “cuproenzymes”), including transcriptional regulators, chaperones, oxidoreductases, free radical scavengers and immune function modulators [49, 50–52, 141]. The adult body contains 50–120 mg of copper, mostly in muscle and the liver. Copper is absorbed in the intestine and released by the liver into bile to prevent copper toxicity [142]. Copper is extremely toxic in excess as it can destabilize Fe/S clusters [143] and increase the generation of free radicals [144]. Mutations in ATP7A or ATP7B disrupt the homeostatic copper balance, resulting in inherited autosomal recessive disorders causing copper deficiency (Menkes disease) or copper overload (Wilson's disease), respectively [145, 146, 147]. Copper deficiency can lead to problems with connective tissues, muscle weakness, anemia, low white blood cell count, immune dysfunction and neurological problems [53]. According to an analysis of data from the 2009–2012 National Health and Nutrition Survey (NHANES), 6%–15% of adults have copper intake lower than recommended [148]. However, copper deficiency is uncommon in humans except in special cases, such as individuals with celiac disease or older people with cachexia [149].

Intracellular copper deficiency increases lipoprotein peroxidation, impairs nitric oxide (NO)‐mediated endothelial dilatation and increases the risk of cardiomyocyte oxidative damage [15]. In the mitochondria, copper is an essential component of complex IV, also known as Cytochrome C oxidase (CCO) [51]. Although copper is crucial for cytochrome oxidase function, the transporters and regulators of mitochondrial copper are unknown. In rats, a copper‐deficient diet caused a 74% decrease in CCO and increased manganese superoxide dismutase (MnSOD) and GPX in isolated heart mitochondria [150, 151]. This is an indirect indication that mitochondrial ROS production was increased [151]. Moreover, specifically cardiac mitochondrial ETC function was compromised in copper‐deficient rats [55]. Additionally, a study in mice by Elsherif et al. [152] showed that early life copper deficiency in mice leads to systolic and diastolic dysfunction in association with histopathological changes in the murine heart. The changes observed were suggested to show the transition from copper deficiency–induced hypertrophy [153] to CHF [152].

Higher blood concentrations of copper have been found in patients with HF compared to healthy controls [154]. Málek et al. [155] reported a possible association between copper status and prognosis in 64 patients with HF. Patients who died or were hospitalized for HF in the following 12 months had, on average, a higher serum copper concentration than those who did not (121 vs. 104 μg/dl [19.0 vs. 16.3 μmol/L]; *p* < 0.001). A meta‐analysis comprising 13 studies including 1504 subjects identified an association between high serum copper and HF [156]. High serum copper concentration in HF may reflect an increase in serum ceruloplasmin, which binds up to 95% of serum copper [157]. Ceruloplasmin plays multiple roles in copper transportation, coagulation, angiogenesis, oxidative stress defense and iron homeostasis [158], and it has also been associated with cardiovascular disease in clinical studies [159]. Ceruloplasmin oxidizes Fe(II) to Fe(III) (ferroxidase) in order for it to be incorporated into transferrin, exerts antioxidant GPX activity, scavenges ROS [44] and may play a fundamental role in protection from iron‐mediated free radical injury [160]. It has also been suggested that the increase in serum copper in HF is due to copper efflux from the myocardium, related to homocysteine dynamics, creating local/tissue deficiency [161]. Of note, ceruloplasmin constitutes an acute phase reactant [162]. Therefore, an increase in serum copper and ceruloplasmin concentrations could also be the consequence of (sub‐)clinical inflammation and stress, but not necessarily the cause. However, the increase of serum copper, and secondarily of ceruloplasmin caused by myocardial infarction, seems not to be completely associated with an inflammatory response [163].

To our knowledge, there is no evidence to support copper supplements for patients with HF. Currently, patients with HFrEF (*n* = 200) are being recruited for a randomized trial to evaluate the effects of a copper‐binding agent called INL1 in patients with HF (TRACER‐HF) [54]. It is hypothesized that INL1 serves as a copper redistribution shuttle to transport copper from the high‐concentration gradient, for example, the circulation, to copper‐depleted tissues such as ischemic myocardial tissue.

## Coenzyme Q10

CoQ10, also known as ubiquinone, is found predominantly in meat, fish and nuts. Although CoQ10 is a common component of most cell membranes, it plays a key role in the mtETC to facilitate ATP production [57]. Mitochondria, and therefore CoQ10, are present in high concentrations in the myocardium, liver and kidneys. In addition, CoQ10 protects the cell membrane against oxidation and inhibits the peroxidation of lipids and lipoproteins [56]. The oxidative stress associated with HF increases the stress on antioxidant systems [164], which may deplete CoQ10 [165, 166, 167].

CoQ10 facilitates electron transfer from complex I (NADH coenzyme Q reductase) to complex III (cytochrome bc1 complex), and from complex II (succinate dehydrogenase) to complex III [58, 59]. It may also stabilize the mitochondrial permeability transition pore and reduce apoptotic cell loss [58]. In vitro experiments show that CoQ10 supplements inhibit low‐density lipoprotein oxidation to a greater degree than other natural antioxidants, such as β‐carotene or α‐tocopherol [168, 169].

In patients with HF, lower plasma concentrations of CoQ10 are associated with poorer NYHA functional class, lower LVEF and higher plasma concentrations of NT‐proBNP [61, 62]. Furthermore, patients with more severe HF (classes III and IV) have lower plasma and myocardial levels of CoQ10, suggesting greater deficiency as the disease worsens [165, 166, 167]. In unadjusted analyses, observational studies suggest that low plasma concentrations of CoQ10 predict a higher mortality in patients with HF [61, 166, 167], although not independently of other prognostic variables. Statins reduce plasma CoQ10 concentrations, but this does not appear to account for the lack of clinical benefit of statins in advanced HF [61, 170, 171]. Most of the CoQ10 in plasma is carried in lipoproteins, the concentration of which may fall with worsening disease severity or statin therapy [57, 172].

Over the past few decades, several randomized trials of CoQ10 supplements in HF have failed to produce conclusive evidence. One of the earliest placebo‐controlled randomized trials studied the effects of 12‐month treatment with CoQ10 in patients with chronic CHF (*n* = 641) [173] and reported fewer HF‐related hospitalizations in those assigned to CoQ10. More recently, between 2003 and 2010 the Q‐SYMBIO enrolled 420 patients in 17 centers internationally [63]. CoQ10 added to standard HF therapy did not improve NYHA functional class, exercise capacity or NT‐proBNP at 16 weeks, but in the longer term (median follow‐up 106 weeks) reduced the primary (composite) endpoint of hospitalization for worsening HF, cardiovascular death, mechanical assist implantation or urgent cardiac transplantation (HR: 0.50; 95% CI: 0.32−0.80; *p* = 0.003). All‐cause mortality was also reduced (HR: 0.51; 95% CI: 0.30−0.89; *p* = 0.018). The KiSel‐10 trial also suggested that a combination of CoQ10 and selenium reduced cardiovascular mortality [108]. Many other, smaller trials have evaluated the effects of CoQ10, and generally found improvements in symptoms, exercise capacity and quality of life [6, 174–180]. However, the heterogeneous populations and study outcomes, different trial designs and follow‐up duration, and different preparations of CoQ10 contribute to the uncertainty of evaluating and pooling data. Furthermore, coenzyme Q10 in its native form may never reach the mitochondria after ingestion, which brings up the question of the actual mode of action of the compound considering the performed clinical studies. Mito‐Q was developed to be able to reach the mitochondria, but has shown negative effects, since the DTPP moiety causes swelling and depolarization of mitochondria [181] and was found to be associated with a decrease in Δψ m and mtDNA copy number [182]. Therefore, more in‐depth mechanistic studies are needed to assess the actual mode of action of the compound in the heart, and in parallel, to develop safe compounds that enable shuttling of coQ10 into mitochondria. Therefore, well‐designed, adequately powered trials are necessary to assess the effect of CoQ10 supplements on the well‐being and survival of patients with HF.

## Multi‐micronutrient interventions

(Mal‐)nutrition of micronutrients has great impact on the human heart and especially on its ability to recover from damage, and consequently associates with prognosis. Improper micronutrient intake is frequently observed in HF patients, affecting 30%–50% of this population [5, 7]. Deficiencies in multiple micronutrients—such as vitamin A, calcium, magnesium, selenium (Se), zinc, iron, vitamin D and iodine—have been documented, without establishing a causative association between them and the onset of HF [183]. The HFSA provided valuable recommendations on diet and nutrition in its most recent guidelines [11]. Adjustment of nutritional status and energy supplementation are recommended in patients with advanced HF. It was also suggested that daily evidence‐based multi‐micronutrient supplementation should be considered for all patients with HF, particularly those receiving diuretic therapy or restricted diets [11].

Several small, double‐blind, randomized trials have evaluated the role for micro‐ and macronutritional supplementation in patients with HFrEF and ischemic heart disease [47, 48, 184]. A first trial enrolled 30 patients and randomized them to placebo or a micronutrient cocktail composed of zinc, selenium, thiamine, CoQ10 and other micronutrients. After 1 year, mean LVEF increased from 26% to 31% (*p* < 0.05) in the group assigned to receive micronutrients but not in those assigned to placebo. However, there was no improvement in NYHA classification in the patients receiving micronutrient therapy [47]. A second trial enrolled 38 patients and randomized them to placebo or a micronutrient cocktail. Among those assigned to supplements, left ventricular end‐diastolic volume shrank by about 5%, but LVEF did not improve [48]. A third trial involving 74 patients with chronic stable HF that assessed the effect of multi‐micronutrient supplementation showed no significant difference in LVEF, nor in quality of life, exercise capacity or biomarkers [184]. A fourth study included 41 patients who underwent coronary artery bypass surgery with an LVEF less than 40% and were randomly assigned to a double‐blind trial of MyoVive supplement or placebo. Here, supplementation was associated with a reduction in left ventricular end‐diastolic volume [48]. Although small, these trials suggest a possible beneficial effect that requires further, more thorough investigation, especially regarding the inconsistent combinations of micronutrients in these studies.

One important factor that is hampering consistency of study results is the supplementation of subjects who are nondeficient (for one or multiple micronutrients). As an example, selenoprotein activity, and therewith supplementation effectiveness, reaches plateau levels at serum selenium concentrations of approximately 125 μg/L, under a sufficiently high selenium supply [185, 186]. This implies that (multi‐)micronutrient supplementation should be assessed in a personalized, nutrient‐specific manner, since supplementing patients sufficient of certain micronutrients has limited effect (select study). A second factor that may influence the results is the geographical location of the included studies, since dietary intake of, for example, selenium is high in Canada, the United States and Japan (>100 μg/day), and much lower in some parts of Europe (∼40 μg/day) [95]. As such, selenium‐replete patients may show less benefit. Supporting this, Kuria et al. reported in a subanalysis a significant reduction of cardiovascular mortality in studies conducted with selenium in Asia (RR: 0.59, 95% CI: 0.45–0.79) and Europe (RR:0.55, 95% CI: 0.45–0.68), but not in the United States (RR: 0.93; 95% CI: 0.82–1.05) [118]. Third, there is a lack of controlled conditions regarding participants’ diet and lifestyle, which can severely impact the study outcome. For instance, many investigations estimate participants’ diet information on memory‐based methods with low scientific rigor, or participants are not advised to follow any diet recommendation [187]. As an example, minerals and/or vitamin supplementation in combination with low‐fat diets (or fasting) drastically limits micronutrient absorption. Particularly, fat‐soluble CoQ10 is not efficiently absorbed. Therefore, micronutrient supplementation should preferably occur in combination with offering balanced meals, or individualized dietary guidance or supplementation to patients with HF [188, 189, 190].

## Conclusions

The failing myocardium might be “an engine out of fuel.” However, increasing the delivery of energy substrates (e.g., fatty acids, glucose, ketones) to the mitochondria [191] cannot succeed if the mitochondria cannot turn these energy substrates into fuel (ATP) without wrecking the underperforming engine (increased ROS). Micronutrient deficiency changes the paradigm from “an engine out of fuel” to “a defective engine on a path to self‐destruction.”

## Conflict of interest

The authors declare that there is no conflict of interest.

## Author contributions

Nils Bomer: conceptualization (lead); methodology (lead); supervision (lead). Mario G. Pavez‐Giani: conceptualization (equal); methodology (supporting). Niels Grote Beverborg: conceptualization (supporting). Peter van der Meer: conceptualization (supporting); supervision (supporting).
